# Exploration and Exploitation of Novel SSR Markers for Candidate Transcription Factor Genes in *Lilium* Species

**DOI:** 10.3390/genes9020097

**Published:** 2018-02-14

**Authors:** Manosh Kumar Biswas, Ujjal Kumar Nath, Jewel Howlader, Mita Bagchi, Sathishkumar Natarajan, Md Abdul Kayum, Hoy-Taek Kim, Jong-In Park, Jong-Goo Kang, Ill-Sup Nou

**Affiliations:** 1 Department of Horticulture, Sunchon National University, 255 Jungang-ro, Suncheon, Jeonnam 57922, Korea; mkbcit@ymail.com (M.K.B.); ujjalnath@gmail.com (U.K.N.); jewel.howlader81@gmail.com (J.H.); bagchi_econ@yahoo.com (M.B.); sathisbioinfo@gmail.com (S.N.); kayumagb@gmail.com (M.A.K.); jipark@sunchon.ac.kr (J.-I.P.); jgkang@sunchon.ac.kr (J.-G.K.); 2 Department of Genetics and Plant Breeding, Bangladesh Agricultural University, Mymensingh-2202, Bangladesh; 3 University-Industry Cooperation Foundation, Sunchon National University, 255 Jungang-ro, Suncheon, Jeonnam 57922, Korea; htkim@sunchon.ac.kr

**Keywords:** transcription factor, SSR markers, genetic diversity, *Lilium* species

## Abstract

Lilies (*Lilium* sp.) are commercially important horticultural crops widely cultivated for their flowers and bulbs. Here, we conducted large-scale data mining of the lily transcriptome to develop transcription factor (TF)-associated microsatellite markers (TFSSRs). Among 216,768 unigenes extracted from our sequence data, 6966 unigenes harbored simple sequence repeats (SSRs). Seventy-one SSRs were associated with TF genes, and these were used to design primers and validate their potential as markers. These 71 SSRs were accomplished with 31 transcription factor families; including bHLH, MYB, C2H2, ERF, C3H, NAC, bZIP, and so on. Fourteen highly polymorphic SSRs were selected based on Polymorphic Information Content (PIC) values and used to study genetic diversity and population structure in lily accessions. Higher genetic diversity was observed in Longiflorum compared to Oriental and Asiatic populations. Lily accessions were divided into three sub-populations based in our structure analysis, and an un-rooted neighbor-joining tree effectively separated the accessions according to Asiatic, Oriental, and Longiflorum subgroups. Finally, we showed that 46 of the SSR-associated genes were differentially expressed in response to *Botrytis elliptica* infection. Thus, our newly developed TFSSR markers represent a powerful tool for large-scale genotyping, high-density and comparative mapping, marker-aided backcrossing, and molecular diversity analysis of *Lilium* sp.

## 1. Introduction

Lily (*Lilium* sp.) is one of the most important commercially-cultivated flowering plants and is widely grown in temperate and sub-tropical regions, including France, Chile, the USA, Japan, New Zealand, and the Netherlands, which is the world’s leading producer and exporter [1]. Lily is a perennial monocotyledon belonging to the family Liliaceae, with the genus *Lilium* including more than 100 species distributed among seven sections (*Archelirion*, *Leucolirion*, *Lilium*, *Martagon*, *Oxypetala*, *Pseudolirium*, and *Sinomartagon*) [2,3,4]. *Lilium* species have a huge genome of 36 Gb and 2n = 2x = 24 chromosomes. Species belonging to the same section are cross-compatible and produce fertile hybrids [2]. They are also highly heterozygous due to a series of interspecies crosses [2]. However, precise measurement of the heterozygosity within *Lilium* remains difficult due to the limited number of molecular markers available to characterize such diversity.

Improvement of lily accessions through conventional breeding is time-consuming, tedious, and cost-intensive due to the long time requirement (averaging three years) from seed-to-flower production [5]. Moreover, most lily cultivars are susceptible to many diseases, although a few commercial lily cultivars are resistant to *Botrytis elliptica*, *Fusarium oxysporum*, and LMoV (Lily mottle virus) [6]. *B. elliptica* is a phytopathogenic fungus that causes necrosis and blight symptoms in lily leaves and is recognized as a devastating disease both in field and greenhouse cultivation, and even during storage and transport [7]. However, it is difficult to develop cultivars resistant against this disease through conventional breeding due to the horizontal nature of resistance reactions controlled by several genes [8]. Therefore, detection of quantitative trait loci (QTLs) and single nucleotide polymorphisms (SNPs) and their utilization in marker-assisted breeding programs might be effective for developing resistant lily cultivars.

Several attempts have been undertaken to utilize molecular markers in lily improvement programs; for example, using Restriction Fragment Length Polymorphisms (RFLPs) to trace the parents of hybrids [9], RAPD (Randomly Amplified Polymorphic DNA) for genetic fidelity tests and diversity analysis [10,11], Inter Simple Sequence Repeats (ISSRs) to detect mutants [12], Simple Sequence Repeats (SSRs) to assess gene flow among populations [13,14], and SNPs to identify suitable loci to construct a mapping population [2]. In addition, 3329 expressed sequence tags (ESTs) are deposited in a public database [15] and EST-derived SSRs were developed for the *Lilium* genus [16]. However, a high-coverage genome sequence with good marker maps is still needed for accurate trait mapping and to initiate marker-assisted breeding. Shahin et al. [8] constructed a genetic linkage map for lily using three types of molecular markers (AFLP—Amplified Fragment Length Polymorphism, DArT—Diversity Array Technology, and NBS—Nucleotide Binding Site profiling) for important ornamental and disease resistance traits but these markers are not sufficiently saturated [8]. Therefore, it is essential to develop highly polymorphic, user-friendly, cost-effective molecular markers for *Lilium* sp.

We reasoned that codominant SSR markers based on transcription factor (TF) genes could serve as useful tools for QTL mapping and marker-assisted breeding of *Lilium* sp. A substantial amount of evidence is available on the role of functional domains in TFs, which act as master regulators controlling different cellular processes, including stress responses, with particular importance in the domestication of monocot and dicot species [17,18].Functional domains of TF genes have been successfully used to develop SSR markers in Solanaceous plant species, such as tomato and pepper [19].However, relatively few SSR markers are available in lily compared to other horticultural crops such as citrus [20,21,22,23], banana [24], and cucumber [25]. One set of SSR markers was developed from ESTs of the *Lilium* sp. [26]. In addition, Yuan et al. [27] developed 1072 SSR primers from *Lilium regale* ESTs, among which 494 were verified by polymerase chain reaction (PCR) and 172 are publicly available [28]. Horning et al. [13] developed six SSR markers for a population of *Lilium philadelphicum*, and 19 polymorphic SSR markers were developed by Lee et al. [16]. Advances in sequencing technologies now provide sufficient information for us to easily and cost-effectively generate transcriptome sequences to develop candidate gene-based genomic resources, such as TF gene-based molecular markers.

Despite this progress, the number of robust, polymorphic, and publicly available SSR markers remains insufficient for many downstream breeding applications in lily; for example, genotyping, cultivars identification, genetic diversity estimation, positional cloning, trait-tagging, and comparative mapping. To boost the breeding program of this valuable crop species, functional molecular markers need to be developed. In this study, we aimed to develop transcription factor gene-associated SSR markers (TFSSRs) by trimming 0.14 Gb RNA-seq data derived from *B. elliptica*-treated *Lilium formolongi* cv. ‘Sinnapal’. We validated the resulting TFSSR markers to characterize 39 lily accessions based on their purity, genetic diversity, and population structure.

## 2. Materials and Methods

### 2.1. Preparation of Plant Materials

For transcriptome sequencing, a cDNA (complementary DNA) library was constructed from *B. elliptica*-infected leaf tissue of *L. formolongi* cv. ‘Sinnapal’. Bulbs of ‘Sinnapal’ lily were planted in plastic pots containing sterilized potting mixture and kept in a greenhouse at 22 °C under 12/12-h light/dark conditions. *B. elliptica* was cultured on petri dishes containing potato dextrose agar (PDA) medium and incubated at 20 °C under UV light for 10 days. Spores were collected by flooding the culture with sterile Tween-20 solution (0.05% Tween-20 in sterilized distilled water) and removing the spores from the hyphae with the help of a sterile glass spreader. The conidia were filtered through four layers of sterile Miracloth (Cat: 475855-1R, Calbiochem^®^, Billerica, MA, USA) to remove any hyphal fragments presented in the spore solutions. Spore concentration was measured using a hemocytometer and adjusted to 5 × 10^4^ spores/mL with sterile distilled water; the resulting spore suspension was stored at 4 °C until use. Leaves of 120-day-old plants were inoculated with *B. elliptica* spore suspension by complete dipping of the leaves for 10 min. At five time points (0, 1, 2, 5, and 7 days), leaf samples were collected from three plants, immediately frozen in liquid nitrogen, and stored at −80 °C until RNA extraction.

### 2.2. cDNA Library Construction

Total RNA was extracted from leaf tissue with an RNeasy mini kit (Qiagen, Hilden, Germany) according to the manufacturer’s instructions; RNA quality and quantity were estimated using 1.5% agarose gel electrophoresis and a NanoDrop spectrophotometer (ND1000, NanoDrop Technologies, Wilmington, DE, USA), respectively. Equal quantities of high-quality RNA from the three leaf samples per time point were pooled together for library preparation and sequencing. cDNA libraries were constructed using the total RNA and converted into libraries of template molecules suitable for subsequent cluster generation using the reagents provided in the Illumina^®^ TruSeq™ RNA sample preparation kits v2 (Illumina Inc., San Diego, CA, USA). Briefly, the poly-A containing messenger RNA (mRNA) molecules were purified using poly-T oligo-attached magnetic beads. Then, mRNA was fragmented into small pieces using divalent cations under elevated temperature. Fragmented mRNA was copied into first-strand cDNA using reverse transcriptase and random primers. Second strand cDNA synthesis was carried out using DNA Polymerase I and RNase H. These cDNA fragments were subjected to an end repair process and addition of a single ‘A’ base, then ligated to the adapters. The products were subsequently purified and enriched by PCR to create the final cDNA library (for details please see Supplementary data 1).

### 2.3. Transcriptome Sequencing and Assembling

The constructed library was sequenced by Theragen Bio Institute, Republic of Korea (Suwon, Republic of Korea) using the Illumina HiSeq2000 platform. Raw reads were filtered by FastQC [29] to obtain high-quality reads. Command line tool Trimmomatic (0.32) was used to trim and crop adapter sequences from the reads. Subsequently, high-quality clean reads were assembled using Trinity with default parameters [30]. Finally, 90,115 transcriptome sequences were generated. In addition, 179,988 and 85,647 transcriptome sequences from two other lily sub-species *Lilium longiflorum* (Easter lily) and *L. longiflorum* (White tower lily) were assembled into non-redundant lily unigenes using CAP3 (for more detail transcriptome sequencing and assembly please visit the site [31]). The transcriptome sequences of *L. longiflorum* (Easter lily) and *L. longiflorum* (White tower lily) were collected from our lily database [31]. Assembled unigene sequences were deposited in the Lsat web server [32] and also into the National Center for Biotechnology Information (NCBI) under Accession No. PRJNA388718.

### 2.4. TFSSR Marker Development and In Silico Characterization

To develop TFSSR markers from lily TF genes, SSRs were identified using Lsat web servers [32]. Lsat is an online SSR analysis tool for lily transcriptome data. We used default parameters of Lsat for the SSR analysis, and the output of the SSR results were downloaded from the Lsat web server. SSR-containing transcriptome sequences were BLAST searched against the Plant Transcription Factor Database version 3.0 (http://planttfdb.cbi.pku.edu.cn/index.php) using an *E*-value cut off of 10^−10^. A Perl script was used to extract SSR motifs containing TF-encoding lily transcript sequences (Supplementary data 2). To filter SSRs containing TF-encoded transcripts, we set a cut-off value of 65% for query coverage with 40% identity. Each extracted transcript was used to design TFSSR primers. The batch mode version of Primer3 [33] software was included in the Perl-based primer design pipeline, and default parameters of Primer3 software were used for TFSSR primer design. TFSSR markers were categorized into different groups according to SSR attributes (as repeat motif length). For example, TFSSRs were classified as Class I (≥20 nt) and Class II (12–20 nt). Based on repeat unit length, TFSSRs were categorized as dinucleotides to hexanucleotides. In terms of SSR motif nucleotide base composition, TFSSRs were classified into three groups: AT-rich, GC-rich, and AT/GC-balanced.

### 2.5. In Silico TFSSR Expression Profiling in Response to Biotic Stress

The expression level of each unigene was quantified by FPKM (Fragments Per Kilobase of transcript per Million mapped reads), which was also used to estimate the fold-change values. All FPKM values were deposited into the Lily database [31]. The FPKM value of each TFSSR marker was searched from the lily database using the unigenes ID (identity) of each TFSSR. A heat map was drawn of the TFSSR by Cluster 3.0 [34] using hierarchical clustering methods.

### 2.6. DNA Extraction from Plant Materials for Validation of TFSSR

Initially, eight lily genotypes from three major groups, viz. Asiatic, Longiflorum, and Oriental, were used to amplify all 71 TFSSR specific primers and assessed for polymorphism. Among them, 14 TFSSRs were selected based on high polymorphism to estimate the genetic diversity, population structure, and phylogenetic relationships by taking 39 lily accessions (Table S1) along with the initially used eight genotypes. All the genotypes were collected from the National Institute of Horticultural and Herbal Science; Korea National College of Agriculture and Fisheries, and Gangwondo Agricultural Research and Extension Services, South Korea. Fresh, green, young leaves of each genotype were collected, immediately frozen in liquid nitrogen, and stored at −80 °C until further use. Total genomic DNA was extracted using the Qiagen DNeasy Plant Mini Kit (QIAGEN, Hilden, Germany) according to the manufacturer’s instructions. The quality and quantity of isolated DNA was checked using agarose gel electrophoresis (1%) and Nanodrop spectrophotometry (Thermo Scientific, Delaware City, DE, USA), respectively. Each DNA sample was diluted to 50 ng/µL for PCR.

### 2.7. Experimental Validation of TFSSRs

All TFSSR markers were validated by PCR amplification followed by 1.5% agarose gel electrophoresis and analysis with a QIAxcel automated DNA fragment analyzer (QIAxcel Advanced, SN: 15268, QIAGEN, Hilden, Germany). PCR amplification was carried out in a total reaction volume of 20 µL, comprising 50 ng genomic DNA, 1× Taq polymerase buffer, 2 mM MgCl_2_, 0.2 mM dNTP mix, 10 mM each of the forward and reverse primers, and 1 U Taq polymerase. PCR amplification was executed in a Takara PCR thermal cycler (TAKARA, Tokyo, Japan) with the following conditions: 94 °C for 5 min, 35 cycles at 94 °C for 30 s, 56–60 °C (according to primer annealing temperature) for 30 s, and 72 °C for 45 s, followed by a final elongation at 72 °C for 5 min. PCR products were separated on 1.5% agarose gels in 1× Tris-Borate-EDTA (TBE) buffer for 30 min at 100 V to estimate amplicon size and PCR specificity. DNA bands were visualized by blue mango (Hipurebio Inc., Daejeon, Korea) staining under UV light, and a 100-bp molecular ladder was used to estimate the amplicon size. PCR products of selected primer pairs were further analyzed in a QIAxcel automated DNA fragment analyzer to estimate polymorphism.

### 2.8. Data Analysis

Amplicon fragment sizes of the tested lily genotypes were calculated using QIAxcel Screen Gel software, version 1.2.0. These data were used to calculate marker amplification frequency and polymorphism level. The Polymorphism Information Content (PIC) was estimated using the formula PIC = Σ*p_i_*^2^, where *p_i_* is the proportion of the *i*^th^ allele. PIC values and allele frequencies were calculated using Power Marker software [35]. SSR genotyping data were used for estimating observed heterozygosity (Ho), expected heterozygosity (He), and principal coordinate analysis (PCoA) using GenAlEx software version 6.5 [36]. PCoA was based on a dissimilarity matrix and was used for population differentiation. Phylogenetic relationships among accessions was determined based on a genetic distance matrix, and a dendrogram was drawn based on the neighbor-joining (NJ) method using MEGA6 [37]. Furthermore, population structure was analyzed using the Bayesian clustering algorithm of STRUCTURE v2.3.4 [38]. Ten independent runs were carried out for *K* values ranging from 1 to 10 using 20,000 iterations after a burn-in of 200,000 steps assuming an admixture model with allelic frequencies correlated. The optimal *K* value was estimated using the Δ*K* procedure [39] of Structure Harvester v0.6.94 [40]. Molecular Variance (AMOVA) was estimated to quantify the variation within the population, among the genotypes, and also between the populations using GenAlEx software version 6.5 [36].

## 3. Results

### 3.1. In Silico Characterization of TFSSR

A total of 216,768 unigene sequences were screened from cDNA libraries of *L. formolongi* (Sinnapal), *L. longiflorum* (Easter lily), and *L. longiflorum* (White tower lily) transcriptome sequences to identify SSR motifs, and the resulting 6966 SSR motifs were extracted (Table 1). The unigenes that contained SSRs were used in BLAST searches of the Plant Transcription Factor Database (version 3.0) to identify those likely to encode TFs. Seventy-one unigenes were identified as TF-encoding transcripts that contained SSR motifs. Primers for these genes were designed with primer3 (http://www.bioinformatics.nl/cgi-bin/primer3plus/primer3plus.cgi) and found suitable for TFSSR primer modeling (Supplementary data 3). These TFSSRs were grouped into different categories based on their characteristics. Among them, tri-nucleotide repeats were more frequent (47 = 66.20%) compared to other repeat types in terms of nucleotide number; moreover, the tri-nucleotide motifs with CCG and CGC were more frequent than the other types of motifs in terms of nucleotides content (Table 1 and Figure S1a). Repeats and motifs with five- and 15-bp, respectively, were the most frequent (Figure S1b,c). TFSSRs of ≥20 nucleotide (nt) and <20 nt were classified as Class I and Class II SSRs, respectively, with 11 SSRs belonging to Class I and 60 SSRs found in Class II (Table 1).

**Figure 1:**
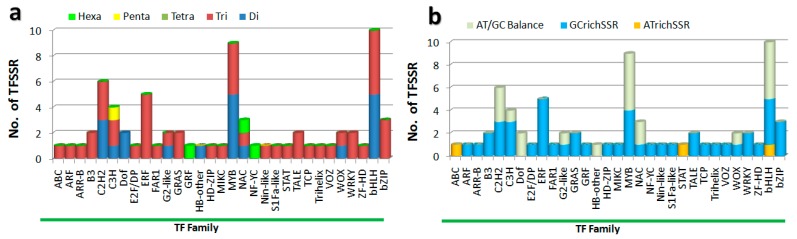
Distribution of TFSSRs in different TF family genes. (**a**) Distribution by family in terms of repeat unit size; (**b**) Distribution by family in terms of motif nucleotide base composition.

SSRs with tri-nucleotide repeats were more frequent than other types of repeats in most of the candidate TF genes. Notably, genes encoding members of the TF families C3H and NAC possessed mixtures of different types of repeats; for example, C3H had di-, tri-, and penta-repeats (Figure 1a). Overall, the 71 SSRs (of Class I and II) were distributed into gene encoding members of 31 different TF families, with 10 SSRs in the bHLH TF family, 9 SSRs in the MYB family, 6 SSRs in the C2H2 family and the rest associated with other TF families (Table S2). Most TF families contained GC-rich SSRs, although ABC and STAT family genes contained AT-rich SSRs (Figure 1b).

### 3.2. TFSSR Expression Profiling upon B. elliptica Infection

**Figure 2:**
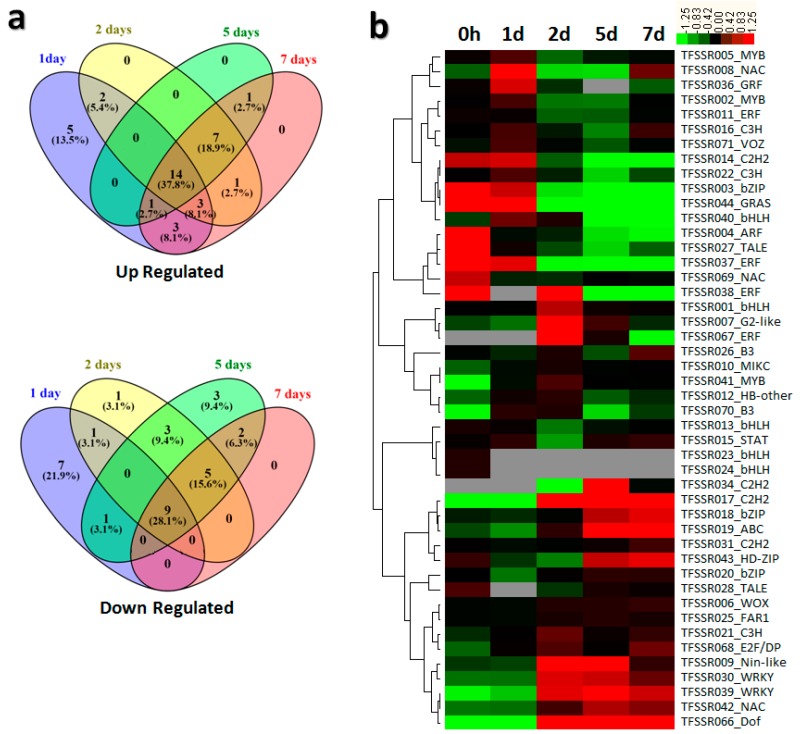
Summary of differential expression of TFSSR-associated genes in response to biotic stress. (**a**) Venn diagram represents time-course specific distribution of TFSSR-associated gene expression among up- and down-regulated categories; (**b**) Heat map showing hierarchical cluster analysis based on the log_2_fragments per kilobase of transcript per million mapped reads(FPKM) values.

A heat map was drawn following hierarchical clustering methods to assess differential expression of TFSSR-associated genes. The 71 TFSSRs were associated with 46 genes that were differentially expressed upon *B. elliptica* infection. Among them, 14 genes were up-regulated and 9 genes were down-regulated across all four time courses (Figure 2a). Considering the gene families, five out of 10 bHLH TF genes were differentially expressed in response to *B. elliptica* infection, and were up-regulated in the early stages (0 h, 1 d) of infection. Four out of six C2H2 TF genes were differentially expressed; one (*TFSSR014*) was up-regulated in the early stages of pathogen infection (0 h, 1 d, 2 d), and the other two (*TFSSR034*, *TFSSR017*) were up-regulated in the later stages of infection (2 d, 5 d, 7 d) (Figure 2b).

### 3.3. Evaluation of Amplification, Polymorphism, and Potential of TFSSRs as Markers

To assess the potential of the 71 TFSSRs as markers, we evaluated the PCR amplification rate, targeted PCR product size, extent of polymorphism, and cross-taxon transferability. The primers for 69 of the TFSSRs successfully amplified products in PCR, with 39 producing amplicons of the expected product size. Out of 69 TFSSRs, 41 produced polymorphic PCR amplicons, while the rest were monomorphic, with an overall average PIC value of 0.58. Together, these 69 TFSSRs markers depicted 207 alleles over the 8 genotypes, with an average of 3.06 alleles per marker and the number of alleles ranging from 2 to 6 (Table 2). The tri-repeats produced the highest number of alleles per locus. Overall, 55 markers were homozygous, and the ratio of homozygous to heterozygous loci was 4:1 (Table 2).

**Figure 3:**
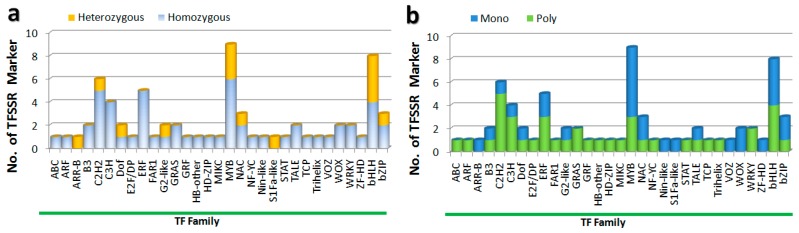
Experimental evaluations of TFSSR markers for 31 TF families. (**a**) Evaluation of homozygosity and heterozygosity by TF family; (**b**) Evaluation of mono and polymorphism by TF family.

We experimentally evaluated our 69 well-amplified TFSSR markers for 31 TF families and found 14 TFSSR markers were heterozygous for 9 TF families, while 55 TFSSR markers were homozygous for 29 TF families. Seven TF families shared both hetero- and homozygous markers (Figure 3a). We also checked efficacy of the 69 SSR markers, which were presented in specific TF-families, to see whether any TF family had an effect on PCR amplifications or not. Almost all markers from the 31 TF families were amplified by PCR, the exceptions being two markers from the bHLH family (Figure S2a). Markers from eight TF families produced non-specific PCR amplicons, while markers from 15 TF-families produced only specific PCR amplicons, and markers from the remaining TF families generated both specific and non-specific PCR products (Figure S2b). Markers from six TF families were only monomorphic, while markers from 15 TF families appeared as polymorphic. The remaining ten TF family markers were a mixture of mono and poly-morphic (Figure 3b). Markers from the C2H2, C3H, and bHLH families were highly polymorphic and their PIC values were larger compared to those of other TF families (Supplementary data 3). We tested the utility of our TFSSR marker to identify hybrids and found 7 of the markers were promising for lily hybrid identification (Figure S3).

### 3.4. Analysis of Genetic Diversity and Population Structure Using TFSSR Markers

Based on polymorphic profiles, a subset of primers covering 14 TFSSRs were randomly selected from the 69 well-amplified TFSSRs for estimation of genetic diversity, population structure, and phylogenetic relationships among 39 lily accessions. These 14 primers detected 113 alleles with an average of 8.07 alleles per marker (Figure S4). The ranges for mean Ho and He were 0–0.62 and 0.28–0.80, respectively, over the population (Table S3). The Ho and Shannon information index (I) values varied within the Asiatic, Longiflorum, and Oriental populations, and they revealed a wide range of variation among the populations even within the same species. Pairwise fixation index (F_ST_) values calculated for population differentiation revealed moderate levels of differentiation among the three populations and also between the populations (Table S4).

**Figure 4:**
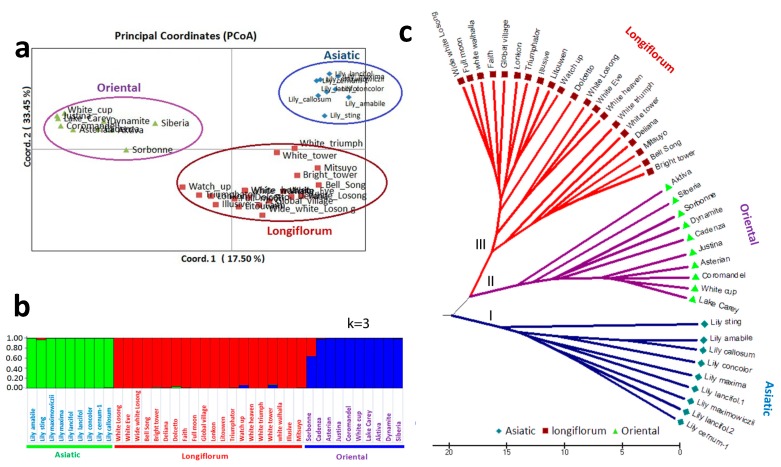
Population structure of Lily germplasm. (**a**) Principal Coordinates Analysis (PCoA) using distance matrix values for 39 lily accessions obtained from primers for 14 TFSSRs, colored circles represent the groups of lily accessions; (**b**) Population structure analysis of 39 lily accessions using STRUCTURE V2.3.4, each vertical bar represents one accession; (**c**) Phylogenetic tree generated by using the variations of PCR amplicon with the 39 lily accessions.

A phylogenetic tree, constructed based on a genetic distance matrix, clustered the 39 lily accessions into three groups (Figure 4a–c). Cluster I contained Asiatic accessions, Cluster II had Oriental accessions, and Cluster III comprised Longiflorum type lilies. In addition, PCoA of the three clusters showed that 17.50% and 33.45% of the variance could be explained by PC1 and PC2, respectively (Figure 4a). AMOVA confirmed the existence of major genetic variation among the individuals compared among populations (Table S5). A Bayesian model-based clustering technique was applied to analyze the genetic variation explained by 14 TFSSR markers. The number of hypothetical populations (*K*) was estimated to elucidate the underlying genetic structure and the maximum delta-*K* value was found using *K* = 3, indicating that the 39 lily accessions belonged to three sub-populations (Figure S5b).

## 4. Discussion

Microsatellite markers are useful for various applications in plant breeding and genetics. Here, we developed SSR markers from putative TF-encoding transcript sequences. To start, we identified SSRs in the lily transcriptome and found that tri-nucleotides occurred most frequently. Similarly, Miao et al. [41], Yuan et al. [28], and Shahin et al. [2] all found higher proportions of tri-repeats in lily EST sequences. The abundance of tri-repeats in the lily transcriptome could be attributed to a selection pressure against frame-shift mutations in transcribed regions, resulting from length changes in non-triplet repeats.

The number of Class II SSRs was almost six-fold higher than that of Class I SSRs in lily TF-gene sequences (Table1). Similar results were also reported in chickpea SSRs mined from TF-encoding transcript sequences [42]. The abundance of Class II SSRs in the transcribed region may be correlated with the length of the transcribed region. In the genomes of higher plants, transcribed regions are generally shorter than the non-transcribed regions; as a result, the frequency of Class II SSRs in the transcribed region might be higher than that of Class I SSRs. Conversely, in terms of the frequency, of transcribed regions, Class I was found to have higher non-transcribed regions than Class II SSRs [21]. In this study, we found the CT motif was abundant among di-nucleotide SSRs, and the CCG motif was the most frequent among the trinucleotide SSRs (Figure S1a). The dominant nature of the CCG tri-motif may be an attribute of monocotyledonous plant species, like lily, as the CCG/CGG motif is also dominant in rice and other monocots [43,44].

In this study, TFSSRs with short repeat numbers and short motif lengths occurred with high frequency (Figure S1b,c). Similar findings have been reported in rice, barley, and tulip [41,45,46]. The occurrence of larger numbers of short repeat motifs and short motif lengths containing SSRs implies that the species may have a relatively rapid rate of evolution. By contrast, species with a large number of repeat and repeat lengths containing SSRs might have a lower mutation rate [41,47]. Hence, we infer from our data that lily TFSSRs may be in the process of rapid evolution.

Taking into account the nucleotide base composition of SSR motifs, TFSSRs can be classified into three groups: AT-rich, GC-rich, and AT/GC-balanced. We found CG-rich SSRs to be more frequent compared to AT-rich SSRs in the lily TF-gene transcriptome. The prevalence of AT-rich or GC-rich SSR loci could be correlated with the GC content of the genome. The GC content of lily TF-gene transcriptome sequences is higher than that of AT, which likely explains the higher proportion of GC-rich SSR loci found in our study, similar to results in banana [24], algae [48], and citrus [21].

Gene expression patterns can provide vital clues for determining gene function, with FPKM values widely used to quantify the expression levels of genes that are differentially expressed across different time points or in different samples. Many studies have demonstrated that TFs control diverse cellular processes and are involved in the responses to abiotic and biotic stresses [17,18,49]. These responses can vary from one plant species to another, and even between diverse genotypes within the same species. Therefore, differentially responsive TF genes may be good candidates for molecular marker development and rapid marker-linked trait identification [42]. To find SSRs associated with TF-encoding genes involved in responses to biotic stress (e.g., *B. elliptica* infection), we took data from differentially expressed genes (DEGs) based on FPKM values corresponding to each TFSSR and performed cluster analysis. Our results revealed that a significant number of TFSSR-associated genes were differentially expressed in response to *B. elliptica* infection. Some genes were up-regulated during the early stages of stress, but then had decreased expression over time courses of stress. By contrast, other genes were down-regulated in the early stages of stress and up-regulated at later stages (Figure 2a). Differential expression patterns of TFSSR-associated genes indicate their likely roles in the response to the *B. elliptica* infection. Thus, our TFSSR markers should be useful for studying the functional diversity of TFSSR-associated genes induced by fungal infection. In addition, TFSSR markers derived from the differentially expressed TF genes may be a good resource for association mapping in combination with the previously published SNPs across the genome of *Lilium* sp. [2].

PCR amplification rate, production of expected PCR amplicons, presence of strong banding patterns, degree of polymorphism, and cross-taxon transferability are the key characteristics typically used to assess the utility of SSR markers. In this study, our TFSSR markers from lily showed high PCR amplification efficiency; similar to TFSSR markers previously reported for lily, citrus, and banana [2,20,21,22,24,25,26,27,28]. The transferability and level of polymorphism of our proposed lily TFSSR markers were higher than those previously reported in lily and other plant species [28]. Usually, the transfer rate and marker polymorphism are associated with the phylogenetic distances among the examined plant species and the existence of variation within the genomic region used for marker development [21]. The proposed lily TFSSR primers exhibited high polymorphism among the populations, therefore, they could be good enough to use as markers. EST- and transcriptome-derived SSRs tend to be more highly conserved than genomic SSRs; therefore, they are more transferable and less polymorphic than genomic SSRs. A high degree of marker transferability in *Lilium* sp. was also reported by Lee et al. [14] and Yuan et al. [16,28].

Markers with high PIC values are useful for genotype identification and estimation of polymorphism at the given loci. In the present study, TFSSR markers with high PIC values reflected their analytical and genotype discrimination power. Hence, our proposed TFSSR markers could be used for seed purity and genetic purity tests of lily hybrids. To assess the performance of TFSSRs in genotype identification, a phylogenetic tree was constructed using 14 TFSSR markers and 39 genotypes; these genotypes were representative of the three main groups of lily (Asiatic, Oriental, and Longiflorum) in accord with the previously reported systematic relationships between *Lilium* sp. [50]. Our data suggest that our proposed markers will be useful in taxonomic, population genetics, and genetic diversity studies, as well as mapping studies in lily and its relatives. We estimated the genetic diversity and population structure using 39 genotypes and found higher than expected He and Ho values, similar to previous results in lily using SSR [50]. SSR markers are well known for detecting higher levels of genetic variation among genotypes or populations compared to other types of molecular markers [51]. Du et al. [26] previously used 57 SSR primers and were able to differentiate 32 lily accessions into two main groups based on their genetic background; however, Du et al. [26] failed to distinguish Asiatic and Longiflorum lily accessions using their SSR markers. Our TFSSR markers were clearly able to distinguish Asiatic, Oriental, and Longiflorum genotypes.

We also used F_ST_ to assess population differentiation based on the genetic polymorphisms of our molecular markers. In this study, the F_ST_ value was 0.297 (Table S3) among three populations, which indicates a moderate level of differences among the three populations. Moderate differences among the lily population were also reported by Lee et al. [51] and Chung and Chung [52]. Our STRUCTURE analysis to distinguish the lily germplasms successfully separated them into three groups, reflecting a major sub-division within the 39 lily germplasm collections and providing further evidence of the utility of our TFSSR markers for lily germplasm characterization.

## 5. Conclusions

SSR markers represent one of the most advanced technologies used to achieve plant breeding goals. Unfortunately, the number of molecular SSR markers for *Lilium* sp. is limited compared to other plant species. In this study, we developed and characterized a novel set of SSR markers from candidate TF-gene transcriptome sequences. We confirmed that these TFSSR markers provide valuable information on the level of polymorphism and diversity in lily. We also identified a set of TFSSR markers that are useful for hybrid identification of lily. Therefore, we recommend the broader application of TFSSR markers, which seem more reliably to characterize lily germplasm compared to other SSR markers. This set of molecular markers may be a powerful and reliable molecular tool to accelerate lily breeding programs.

## Figures and Tables

**Table 1:** In silico characterization of transcription factor simple sequence repeats (SSR) (TFSSR) extracted from transcription factor (TF) sequences of lily for marker development.

Item	Count	%
No. of sequences searched	216,768	
SSR-containing sequences	6966	3.21
Transcription factor SSRs	71	1.99
Di-nucleotide repeats	20	28.17
Tri-nucleotide repeats	47	66.20
Tetra-nucleotide repeats	0	0.00
Penta-nucleotide repeats	1	1.41
Hexa-nucleotide repeats	3	4.23
Class I members	11	15.49
Class II members	60	84.51
GC-rich SSRs	47	66.20
AT-rich SSRs	3	4.23
AT/GC-balanced SSRs	21	29.58

**Table 2:** Evaluation of TFSSR primer pairs for the different repeat classes (based on 8 genotypes).

Parameters	Di	Tri	Tetra	Penta	Hexa	Total/Average
Tested primer	20	47	na	1	3	71
PCR amplification	18	47	na	1	3	69
Band Specific	9	28	na	0	2	39
Scorable Primer	11	31	na	0	2	44
Polymorphic	10	29	na	0	2	41
Range of Alleles No.	2–5	2–6	na	na	2–3	2–6
Total No. of Alleles	50	146	na	4	7	207
No. of Homozygous	12	40	na	1	2	55
No. of Heterozygous	6	7	na	0	1	14
Homo:Hetero Ratio	2:1	6:1	na	na	2:1	4:1
Mean of Alleles ±SD	2.78 ± 0.94	3.11 ± 1.5	na	na	2.33 ± 0.58	3.06 ± 0.755
PIC±SD	0.55 ± 0.17	0.64 ± 0.15	na	0.47 ± 0	0.65 ± 0.16	0.58 ± 0.12

PIC: Polymorphic Information Content; SD: Standard Deviation; na: not available.

## Supplementary Materials

The following are available online at www.mdpi.com/2073-4425/9/2/97/s1, Supplementary data 1: Detailed procedure of cDNA library preparation, sequencing, and assembling; Supplementary data 2: Perl script used to extract SSR motifs containing TF-encoding lily transcript sequences; Supplementary data 3: TFSSR Primer sequences and their characteristics. Figure S1: Distribution of SSR motifs; Figure S2: Evaluation of TFSSR marker based on wet lab towards 31 TF; Figure S3: Banding profile of the 7 TFSSR markers able to identify hybrid crosses between inbreed lines *Lilium lonfiflorum* (Easter lily) L2-4 and *Lilium longiflorum* (Easter lily) L2-28; Figure S4: Allele frequencies by population; Figure S5: Estimated log likelihood of the data. Table S1: List of the plant materials used in this study; Table S2: Distribution for TFSSR markers in different TF families by number count and by percentages; Table S3: Summary of genetic variation statistics for 14 loci; Table S4: Pair wise Fst among populations; Table S5: Summary molecular variance.
