# Supplementary material for: Exploration and Exploitation of Novel SSR Markers for Candidate Transcription Factor Genes in Lilium Species
Source: Genes (Basel). 2018 Feb 14;9(2):97. doi: 10.3390/genes9020097 (PMC5852593; doi:10.3390/genes9020097)
Supplement: Supplementary file 1 [file genes-09-00097-s001.zip › Supplementary materials/Supplementary data1.docx]

**Supplementary data 1: Detail procedure of cDNA library preparation, sequencing and assembling.**

**1. RNA-Seq Experiment**

**1.1 Experiment overview**

The mRNA in total RNA converted into a library of template molecules suitable for subsequent cluster generation using the reagents provided in the Illumina ® TruSeq™ RNA Sample Preparation Kit. The first step in the workflow involves purifying the poly‐A containing mRNA molecules using poly‐T oligo‐attached magnetic beads. Following purification, the mRNA is fragmented into small pieces using divalent cations under elevated temperature. The cleaved RNA fragments are copied into first strand cDNA using reverse transcriptase and random primers. This is followed by second strand cDNA synthesis using DNA Polymerase I and RNase H. These cDNA fragments then go through an end repair process, the addition of a single ‘A’ base, and then ligation of the adapters. The products are then purified and enriched with PCR to create the final cDNA library.

**1.2 Experiment procedure**

**1.2.1 TruSeq mRNA library construction**

**Purify and Fragment mRNA**

This process purifies the poly‐A containing mRNA molecules using poly‐T oligo‐attached magnetic beads using two rounds of purification. During the second elution of the poly‐A RNA, the RNA is also fragmented and primed for cDNA synthesis.

**Synthesize First Strand cDNA**

This process reverse transcribes the cleaved RNA fragments primed with random hexamers into first strand cDNA using reverse transcriptase and random primers.

**Synthesize Second Strand cDNA**

This process removes the RNA template and synthesizes a replacement strand to generate double‐stranded (ds) cDNA. Ampure XP beads are used to separate the ds cDNA from the 2nd strand reaction mix.

**Perform End Repair**

This process converts the overhangs resulting from fragmentation into blunt ends, using an End Repair (ERP) mix. The 3ʹ to 5ʹ exonuclease activity of this mix removes the 3ʹ overhangs and the polymerase activity fills in the 5ʹ overhangs.

**Adenylate 3' Ends A single**

‘A’ nucleotide is added to the 3’ ends of the blunt fragments to prevent them from ligating to one another during the adapter ligation reaction. A corresponding single ‘T’ nucleotide on the 3’ end of the adapter provides a complementary overhang for ligating the adapter to the fragment. This strategy ensures a low rate of chimera (concatenated template) formation.

**Ligate Adapters**

This process ligates multiple indexing adapters to the ends of the ds cDNA, preparing them for hybridization onto a flow cell.

**Enrich DNA Fragments**

This process uses PCR to selectively enrich those DNA fragments that have adapter molecules on both ends and to amplify the amount of DNA in the library. The PCR is performed with a PCR primer cocktail that anneals to the ends of the adapters. The number of PCR cycles should be minimized to avoid skewing the representation of the library

**Enriched Library Validation**

Macrogen perform procedures for quality control analysis on the sample library and quantification of the DNA library templates.

**1.2.2 Clustering & Sequencing**

Illumina utilizes a unique "bridged" amplification reaction that occurs on the surface of the flow cell. A flow cell containing millions of unique clusters is loaded into the HiSeq 2000 for automated cycles of extension and imaging.

Solexa's Sequencing-by-Synthesis utilizes four proprietary nucleotides possessing reversible fluorophore and termination properties. Each sequencing cycle occurs in the presence of all four nucleotides leading to higher accuracy than methods where only one nucleotide is present in the reaction mix at a time. This cycle is repeated, one base at a time, generating a series of images each representing a single base extension at a specific cluster.

**2. Data Handling Procedure**

**2.1 Sequence quality check**

**2.1.1 FastQC**

FastQC aims to provide a simple way to do some quality control checks on raw sequence data coming from high throughput sequencing pipelines. It provides a modular set of analyses which you can use to give a quick impression of whether your data has any problems of which you should be aware before doing any further analysis.

More information can be found here (http://www.bioinformatics.babraham.ac.uk/projects/fastqc).

**2.1.2 Trimmomatic**

Trimmomatic (0.32) is a fast, multithreaded command line tool that can be used to trim and crop Illumina (FASTQ) data as well as to remove adapters. These adapters can pose a real problem depending on the library preparation and downstream application.

More information can be found here (http://www.usadellab.org/cms/?page=trimmomatic).

**2.2 Data analysis**

**2.2.1 Trinity**

Trinity (r20140717), developed at the Broad Institute and the Hebrew University of Jerusalem, represents a novel method for the efficient and robust de novo reconstruction of transcriptomes from RNA-seq data.

More information can be found here (http://trinityrnaseq.sourceforge.net/).

**2.2.2 RSEM-based abundance estimation**

RSEM (1.2.15) is an accurate and user-friendly software tool for quantifying transcript abundances from RNA-Seq data. As it does not rely on the existence of a reference genome, it is particularly useful for quantification with de novo transcriptome assemblies. In addition, RSEM has enabled valuable guidance for cost-efficient design of quantification experiments with RNA-Seq, which is currently relatively expensive.

Trinity also provides their own script for abundance estimation. That script is based on RSEM algorithm. We are using this script for abundance estimation of RNA.

More information can be found here

(http://deweylab.biostat.wisc.edu/rsem/)

(http://trinityrnaseq.sourceforge.net/analysis/align_visualize_quantify.html).

**2.2.3 BlastX (Gene Ontology)**

This program compares the six-frame conceptual translation products of a nucleotide query sequence (both strands) against a protein sequence database (go_v20140820).

More information can be found here

(http://blast.ncbi.nlm.nih.gov/Blast.cgi?CMD=Web&PAGE_TYPE=BlastDocs&DOC_TYPE=ProgSelectionGuide)
