# Supplementary material for: Exploration and Exploitation of Novel SSR Markers for Candidate Transcription Factor Genes in Lilium Species
Source: Genes (Basel). 2018 Feb 14;9(2):97. doi: 10.3390/genes9020097 (PMC5852593; doi:10.3390/genes9020097)
Supplement: Supplementary file 1 [file genes-09-00097-s001.zip › Supplementary materials/Supplementary Figures.pdf]

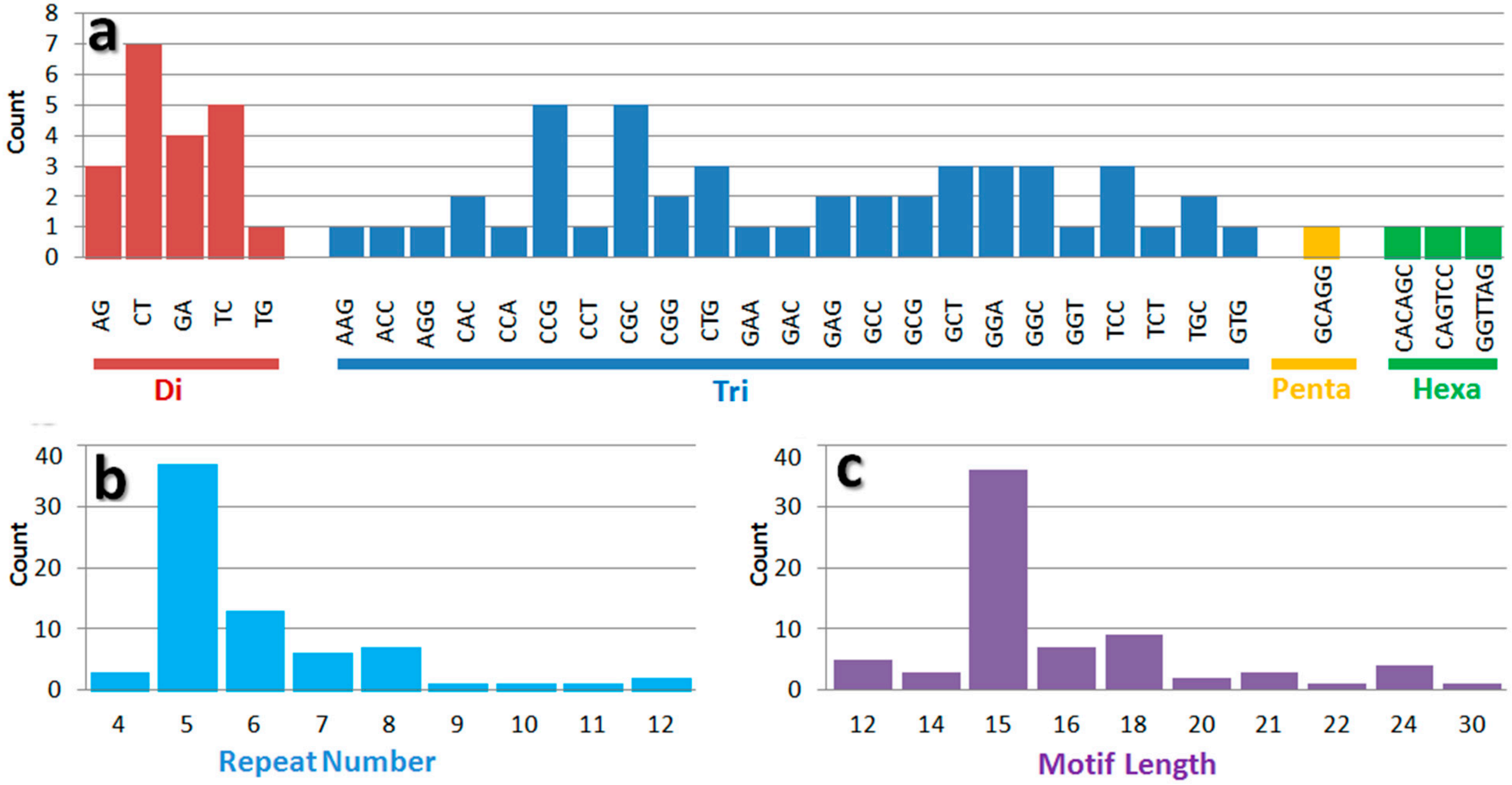

Figure S1. Distribution of SSR motifs: (a) motif types, (b) repeat number, and (c) motif length.

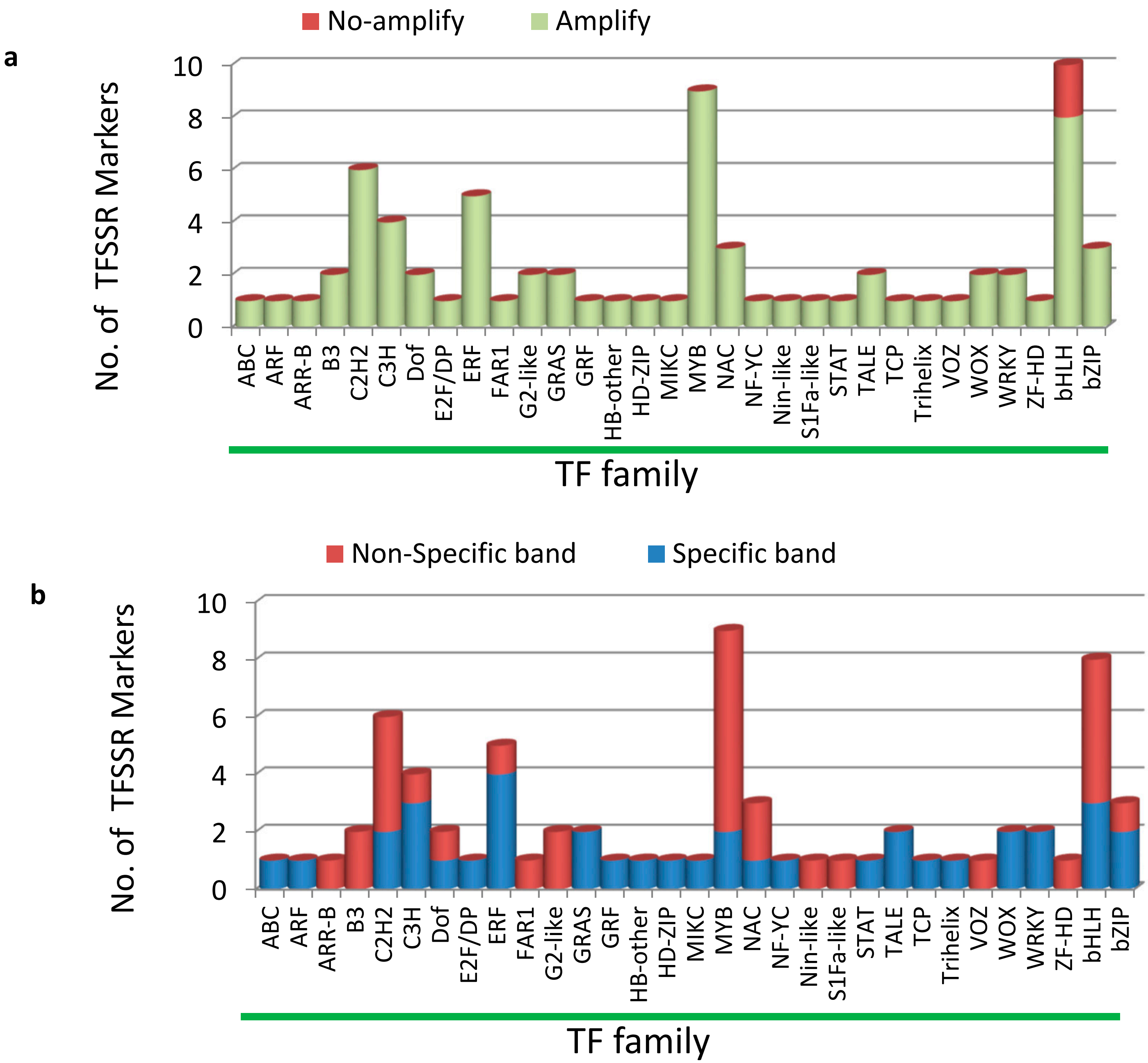

Figure S2. Evaluation of TFSSR markers based on wet lab of 31 TF families (a) based o nPCR amplification and (b) based on band specificity.

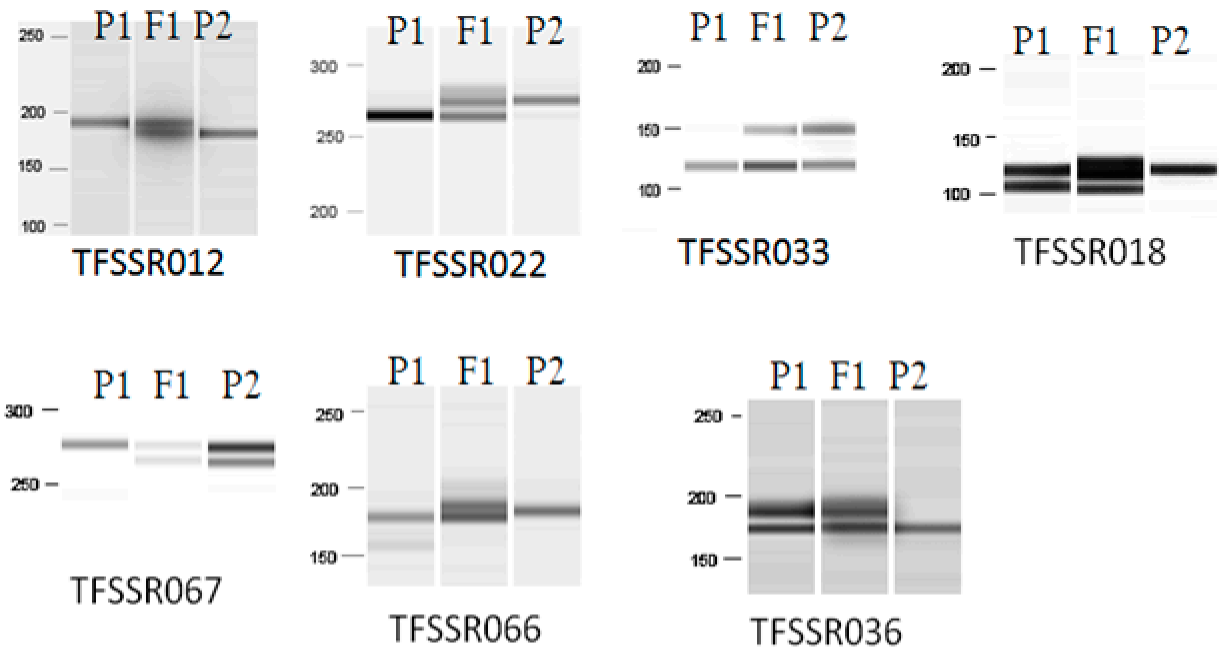

Figure S3. Banding profile of the 7 TFSSR markers able to identify hybrid crosses between inbreed lines *Lilium longiflorum* **(Easter lily)** L2-4 and *Lilium longiflorum* **(Easter lily)** L2-28. [P1= L2-4 P2= L2-28 and F1= L2-4X L2-28].

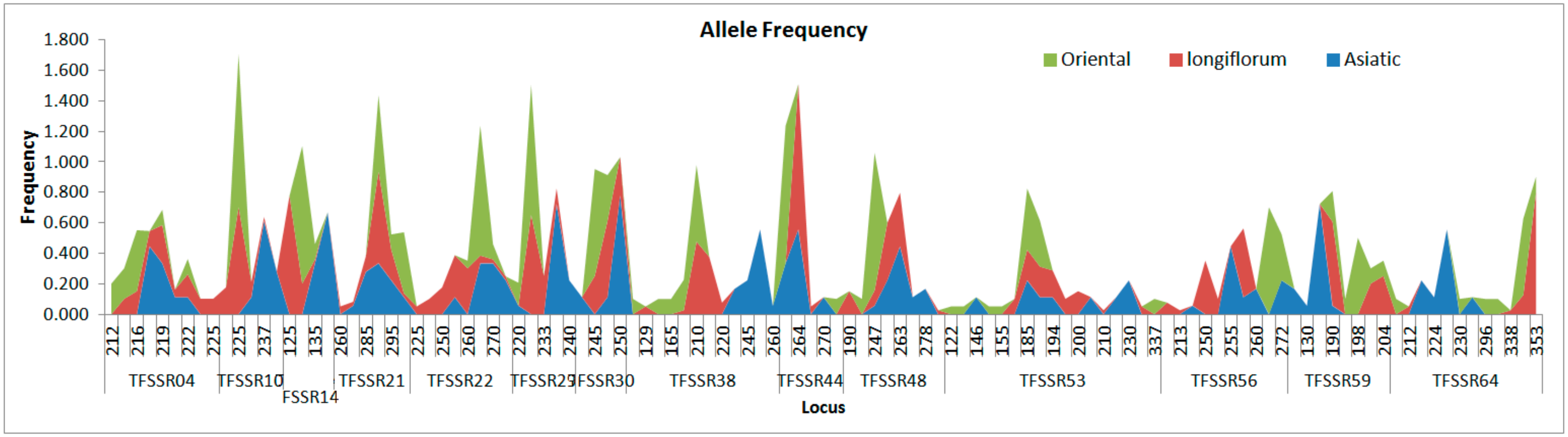

Figure S4. Allele frequencies by population.

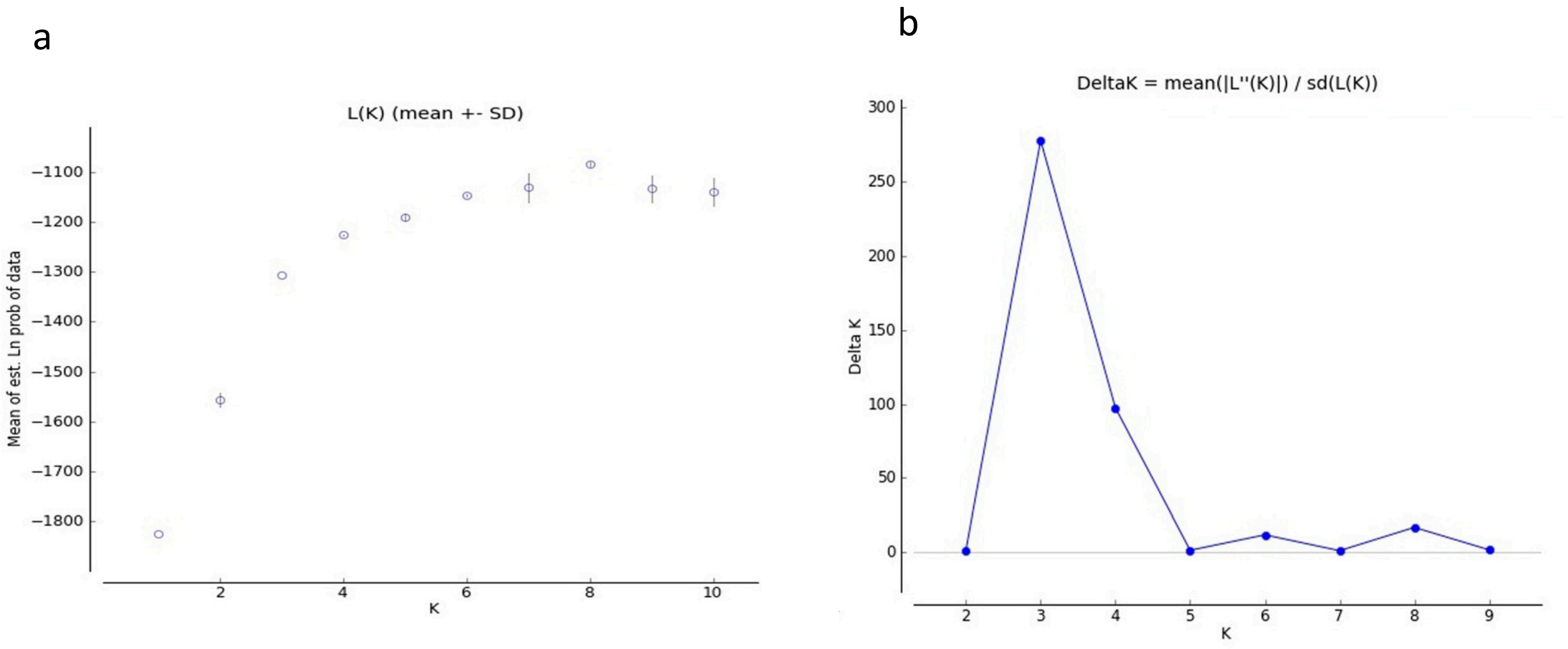

Figure S5. (a) Estimated log likelihood of the data; LnP(D) over 10 repeats of STRUCTURE analysis and (b) the true value of K was obtained following the delta-K method of Evanno et al. [37].
