# Supplementary material for: Exploration and Exploitation of Novel SSR Markers for Candidate Transcription Factor Genes in Lilium Species
Source: Genes (Basel). 2018 Feb 14;9(2):97. doi: 10.3390/genes9020097 (PMC5852593; doi:10.3390/genes9020097)
Supplement: Supplementary file 1 [file genes-09-00097-s001.zip › Supplementary materials/Supplementary Tables.doc]

**Table S1** List of the plant materials used in this study

| No. | Genotype | Group | Scientific name | Sl No | Genotype | Group | Scientific name |
| --- | --- | --- | --- | --- | --- | --- | --- |
| 01 | Lily amabile | Asiatic | *Lilium asiaticum* | 21 | Litouwen | Longiflorum | *Lilium longiflorum* |
| 02 | Lily sting | Asiatic | *Lilium asiaticum* | 22 | Triumphator | Longiflorum | *Lilium longiflorum* |
| 03 | Lily maximowiczii | Asiatic | *Lilium asiaticum* | 23 | Watch up | Longiflorum | *Lilium longiflorum* |
| 04 | Lily maxima* | Asiatic | *Lilium asiaticum* | 24 | White heaven | Longiflorum | *Lilium longiflorum* |
| 05 | Lily lancifol | Asiatic | *Lilium asiaticum* | 25 | White triumph | Longiflorum | *Lilium longiflorum* |
| 06 | Lily lancifol* | Asiatic | *Lilium asiaticum* | 26 | White tower* | Longiflorum | *Lilium longiflorum* |
| 07 | Lily concolor | Asiatic | *Lilium asiaticum* | 27 | white walhalla | Longiflorum | *Lilium longiflorum* |
| 08 | Lily cernum-1 | Asiatic | *Lilium asiaticum* | 28 | Illusive | Longiflorum | *Lilium longiflorum* |
| 09 | Lily callosum | Asiatic | *Lilium asiaticum* | 29 | Mitsuyo | Longiflorum | *Lilium longiflorum* |
| 10 | White Losong | Longiflorum | *Lilium longiflorum* | 30 | Sorbonne* | Oriental | *Lilium orientalis* |
| 11 | White Eve | Longiflorum | *Lilium longiflorum* | 31 | Cadenza* | Oriental | *Lilium orientalis* |
| 12 | Wide white Losong | Longiflorum | *Lilium longiflorum* | 32 | Asterian* | Oriental | *Lilium orientalis* |
| 13 | Bell Song | Longiflorum | *Lilium longiflorum* | 33 | Justina | Oriental | *Lilium orientalis* |
| 14 | Bright tower* | Longiflorum | *Lilium longiflorum* | 34 | Coromandel | Oriental | *Lilium orientalis* |
| 15 | Deliana | Longiflorum | *Lilium longiflorum* | 35 | White cup | Oriental | *Lilium orientalis* |
| 16 | Dolcetto | Longiflorum | *Lilium longiflorum* | 36 | Lake Carey | Oriental | *Lilium orientalis* |
| 17 | Faith | Longiflorum | *Lilium longiflorum* | 37 | Aktiva | Oriental | *Lilium orientalis* |
| 18 | Full moon* | Longiflorum | *Lilium longiflorum* | 38 | Dynamite | Oriental | *Lilium orientalis* |
| 19 | Global village | Longiflorum | *Lilium longiflorum* | 39 | Siberia | Oriental | *Lilium orientalis* |
| 20 | Lonkon | Longiflorum | *Lilium longiflorum* |  |  |  |  |

Note: * represent the name of genotypes use for primary screening of the 71 TFSSR markers.

**Table S2. Distribution of TFSSR markers in different TF family by count of number and by percentages calculation**

| TF Family | TFSSR | Total TF | % |
| --- | --- | --- | --- |
| ABC | 1 | 1 | 1 |
| ARF | 1 | 12 | 1 |
| ARR-B | 1 | 1 | 1 |
| B3 | 2 | 18 | 3 |
| bHLH | 10 | 27 | 14 |
| bZIP | 3 | 13 | 4 |
| C2H2 | 6 | 18 | 8 |
| C3H | 4 | 9 | 6 |
| Dof | 2 | 2 | 3 |
| E2F/DP | 1 | 7 | 1 |
| ERF | 5 | 10 | 7 |
| FAR1 | 1 | 34 | 1 |
| G2-like | 2 | 8 | 3 |
| GRAS | 2 | 39 | 3 |
| GRF | 1 | 4 | 1 |
| HB-other | 1 | 6 | 1 |
| HD-ZIP | 1 | 27 | 1 |
| MIKC | 1 | 6 | 1 |
| MYB | 9 | 63 | 13 |
| NAC | 3 | 48 | 4 |
| NF-YC | 1 | 4 | 1 |
| Nin-like | 1 | 1 | 1 |
| S1Fa-like | 1 | 1 | 1 |
| STAT | 1 | 3 | 1 |
| TALE | 2 | 5 | 3 |
| TCP | 1 | 9 | 1 |
| Trihelix | 1 | 10 | 1 |
| VOZ | 1 | 1 | 1 |
| WOX | 2 | 5 | 3 |
| WRKY | 2 | 13 | 3 |
| ZF-HD | 1 | 3 | 1 |

**Table S3 Summary of Genetic Variation Statistics for 14 Loci**

| **Locus** | **Observed Heterozygosity (Ho)** | | | | **Expected Heterozygosity (He)** | | | | **Shannon Infromation Index (I)** | | | Fst (over all) |
| --- | --- | --- | --- | --- | --- | --- | --- | --- | --- | --- | --- | --- |
|  | **Asiatic** | **Longiflorum** | **Oriental** | **Over all** | **Asiatic** | **Longiflorum** | **Oriental** | **Over all** | **Asiatic** | **Longiflorum** | **Oriental** |  |
| **TFSSR04** | 0.000 | 0.000 | 0.000 | 0.00 | 0.667 | 0.850 | 0.740 | 0.75 | 1.215 | 1.987 | 1.471 | 0.112 |
| **TFSSR10** | 0.333 | 0.250 | 0.000 | 0.19 | 0.537 | 0.469 | 0.000 | 0.34 | 0.901 | 0.877 | 0.000 | 0.457 |
| **TFSSR14** | 0.000 | 0.450 | 0.000 | 0.15 | 0.444 | 0.359 | 0.180 | 0.33 | 0.637 | 0.612 | 0.325 | 0.549 |
| **TFSSR21** | 0.111 | 0.500 | 0.400 | 0.34 | 0.747 | 0.586 | 0.580 | 0.64 | 1.461 | 1.193 | 0.943 | 0.079 |
| **TFSSR22** | 0.222 | 0.750 | 0.100 | 0.36 | 0.716 | 0.788 | 0.265 | 0.59 | 1.311 | 1.736 | 0.518 | 0.230 |
| **TFSSR29** | 0.333 | 0.000 | 0.300 | 0.21 | 0.426 | 0.505 | 0.255 | 0.40 | 0.730 | 0.857 | 0.423 | 0.399 |
| **TFSSR30** | 0.222 | 0.000 | 0.000 | 0.07 | 0.370 | 0.625 | 0.420 | 0.47 | 0.684 | 1.040 | 0.611 | 0.315 |
| **TFSSR38** | 0.556 | 0.900 | 0.400 | 0.62 | 0.611 | 0.625 | 0.680 | 0.64 | 1.120 | 1.158 | 1.359 | 0.226 |
| **TFSSR44** | 0.000 | 0.000 | 0.000 | 0.00 | 0.568 | 0.095 | 0.180 | 0.28 | 0.937 | 0.199 | 0.325 | 0.513 |
| **TFSSR48** | 0.667 | 0.350 | 0.000 | 0.34 | 0.710 | 0.704 | 0.180 | 0.53 | 1.398 | 1.342 | 0.325 | 0.299 |
| **TFSSR53** | 0.222 | 0.350 | 0.200 | 0.26 | 0.840 | 0.844 | 0.730 | 0.80 | 1.889 | 1.936 | 1.557 | 0.062 |
| **TFSSR56** | 0.556 | 0.250 | 0.200 | 0.34 | 0.710 | 0.659 | 0.420 | 0.60 | 1.398 | 1.244 | 0.611 | 0.290 |
| **TFSSR59** | 0.444 | 0.000 | 0.000 | 0.15 | 0.444 | 0.595 | 0.680 | 0.57 | 0.855 | 0.997 | 1.359 | 0.296 |
| **TFSSR64** | 0.000 | 0.300 | 0.000 | 0.10 | 0.617 | 0.341 | 0.700 | 0.55 | 1.149 | 0.680 | 1.498 | 0.325 |
| **Mean** | **0.262** | **0.293** | **0.114** | **0.223** | **0.601** | **0.575** | **0.429** | **0.535** | **1.120** | **1.133** | **0.809** | **0.297** |
| **SE** | **0.02** | **0.02** | **0.01** | **0.01** | **0.01** | **0.01** | **0.02** | **0.01** | **0.03** | **0.04** | **0.04** | **0.01** |

**Table S4** Pair wise Fst among population

| Subspecies | Asiatic | Longiflorum | Oriental |
| --- | --- | --- | --- |
| Asiatic | 0 |  |  |
| Longiflorum | 0.130 | *0* |  |
| Oriental | 0.341 | 0.209 | 0 |

**Table S5 Summary AMOVA**

| Source | df | SS | MS | Estimated Variance |
| --- | --- | --- | --- | --- |
| Among Population | 2 | 102.897 | 51.448 | 1.868 |
| Among Individual | 36 | 231.142 | 6.421 | 2.371 |
| Within Individual | 39 | 65.500 | 1.679 | 1.679 |
| Total | 77 | 399.538 |  | 5.918 |
